# RABL6A, a Novel RAB-Like Protein, Controls Centrosome Amplification and Chromosome Instability in Primary Fibroblasts

**DOI:** 10.1371/journal.pone.0080228

**Published:** 2013-11-25

**Authors:** Xuefeng Zhang, Jussara Hagen, Viviane P. Muniz, Tarik Smith, Gary S. Coombs, Christine M. Eischen, Duncan I. Mackie, David L. Roman, Richard Van Rheeden, Benjamin Darbro, Van S. Tompkins, Dawn E. Quelle

**Affiliations:** 1 Department of Pharmacology, University of Iowa, Iowa City, Iowa, United States of America; 2 The Molecular and Cellular Biology Graduate Program, University of Iowa, Iowa City, Iowa, United States of America; 3 Department of Biology, Waldorf College, Forest City, Iowa, United States of America; 4 Department of Pathology, Microbiology and Immunology, Vanderbilt University Medical Center, Nashville, Tennessee, United States of America; 5 Division of Medicinal and Natural Products Chemistry, University of Iowa, Iowa City, Iowa, United States of America; 6 Department of Pediatrics, University of Iowa, Iowa City, Iowa, United States of America; 7 Department of Pathology, University of Iowa, Iowa City, Iowa, United States of America; University of Hong Kong, Hong Kong

## Abstract

RABL6A (RAB-like 6 isoform A) is a novel protein that was originally identified based on its association with the Alternative Reading Frame (ARF) tumor suppressor. ARF acts through multiple p53-dependent and p53-independent pathways to prevent cancer. How RABL6A functions, to what extent it depends on ARF and p53 activity, and its importance in normal cell biology are entirely unknown. We examined the biological consequences of RABL6A silencing in primary mouse embryo fibroblasts (MEFs) that express or lack ARF, p53 or both proteins. We found that RABL6A depletion caused centrosome amplification, aneuploidy and multinucleation in MEFs regardless of ARF and p53 status. The centrosome amplification in RABL6A depleted p53−/− MEFs resulted from centrosome reduplication via Cdk2-mediated hyperphosphorylation of nucleophosmin (NPM) at threonine-199. Thus, RABL6A prevents centrosome amplification through an ARF/p53-independent mechanism that restricts NPM-T199 phosphorylation. These findings demonstrate an essential role for RABL6A in centrosome regulation and maintenance of chromosome stability in non-transformed cells, key processes that ensure genomic integrity and prevent tumorigenesis.

## Introduction

The centrosome is a small organelle composed of a pair of centrioles and surrounding pericentriolar material (PCM) [1]. One centrosome is inherited by each daughter cell after cell division, duplicated at G1-S, and matures during S-G2 phase so that two centrosomes are present for mitosis. Centrosomes nucleate and anchor microtubules and are required for bipolar mitotic spindle formation, thereby ensuring accurate chromosome segregation during cytokinesis. Centrosome amplification (3 or more centrosomes per cell) is prevalent in solid tumors, and is a primary cause of chromosomal instability (CIN) and accelerated tumor progression [2]–[8].

Centrosome amplification can result from a variety of cellular abnormalities including failed cytokinesis and reduplication of centrosomes during the cell cycle [3]. One common event leading to centrosome overduplication is cyclin-dependent kinase 2 (Cdk2)-mediated hyperphosphorylation of threonine 199 (T199) within nucleophosmin/B23 (NPM) [9], a major regulator of genomic stability [10]. Cells with more than two centrosomes form too many spindle poles (>2) leading to defective chromosome segregation, aneuploidy and/or multi-nucleation (itself arising from failed cell division) [3]. While many cells die due to these defects, those that survive display elevated CIN (abnormal chromosome numbers & structures) and are often highly tumorigenic. Thus, proper centrosome control is vital for maintaining genomic integrity and preventing tumor development and progression [3],[6],[11].

Two of the most commonly inactivated tumor suppressor genes in human cancers are *TP53* and *INK4a/ARF* [12],[13]. Both genes encode key checkpoint regulators, p53 and ARF (Alternative Reading Frame protein), which normally protect the genome by inducing cell death or arrest in response to various cellular stresses [14],[15]. CIN results from loss of functional p53 or ARF. Ablation of p53 in mouse embryo fibroblasts (MEFs) causes robust centrosome amplification and tetraploidy [16]. ARF is a primary activator of p53 [15]; thus, loss of ARF may promote CIN due to reduced p53 function in response to oncogene activation and other cellular stresses. ARF also has numerous anticancer activities that are independent of p53 [14], and it suppresses aneuploidy in MEFs lacking p53 [17].

We discovered a new “Partner of ARF” in a two-hybrid screen using ARF as bait, initially named it Parf (as it was an uncharacterized gene, *c9orf86*), and demonstrated it interacts with ARF’s essential functional domains [18]. Later studies identified Parf from *in silico* analyses based on the presence of a Ras/Rab-like domain in its N-terminus and dubbed the protein RBEL1 [19],[20]. In accordance with current database nomenclature and to underline the fact that Parf/RBEL1 is a member of a family of RAB-like proteins, we have adopted the designation RABL6 for the protein. Four isoforms of RABL6 exist due to alternative splicing [20] and are here named RABL6A-D. All isoforms have GTPase activity [19],[20]. In contrast, only RABL6A contains the carboxy terminal sequences required for ARF binding [18]. RABL6A is the most highly expressed form and it resides in both the cytoplasm and nuclei [18],[19],[21]. Very little is known about RABL6A. Knockdown of RABL6A or all four isoforms was found to cause significant death of various cancer cells [20]–[22], but how RABL6A functions and its activity in normal cells have yet to be determined.

Here, we show that RABL6A is a new regulator of NPM phosphorylation whose depletion in primary fibroblasts promotes both centrosome amplification and CIN. This activity of RABL6A is independent of ARF and p53. These findings identify a novel factor controlling centrosome biology and suggest RABL6A may play an important role in cancer.

## Materials and Methods

### Cell culture

Primary wild-type, *p53*−*/*− and triple knockout (TKO) *p53/Mdm2/ARF*−*/*− MEFs (TKOs kindly provided by Martine Roussel, St. Jude Children’s Research Hospital) [23] were grown in Dulbecco’s Modified Eagles Medium (DMEM) containing 10% fetal bovine serum, 4 mM glutamine, 100 µg/ml penicillin/streptomycin, 0.1 mM non-essential amino acids and 55 µM 2-mercaptoethanol.

### DNA Constructs

The human RABL6A cDNA [18] was shuttled into the pMSCV-IRES-GFP expression vector [24]. Primers (Integrated DNA Technologies, Coralville, IA) for full-length RABL6A are: forward 5′- ccatcgataagatgttttccgccctga -3′; reverse 5′- atatcgatctagagctcctcgtagtcgc -3′. PCR products were directly ligated into pCRII-TOPO (Invitrogen, Carlsbad, CA), sequenced, and subcloned into pMSCV-tk-neo and pGEX-4T-2 vectors. pMSCV-tk-neo constructs containing ARF cDNAs have been described [25],[26]. Recombinant pcDNA3 plasmids containing FLAG-tagged wild-type or T199A substitution mutant of NPM (kindly provided by Kenji Fukasawa, H. Lee Moffit Cancer Center and Research Institute) were digested with BamHI and EcoRI, and inserts subcloned into the pBabe-puro vector.

### RNA Interference and qRT-PCR

Scrambled control and mouse RABL6A targeting short hairpin RNAs (shRNAs) were designed and purchased from Promega (Madison, WI; sequences available on request). Complementary forward and reverse oligonucleotides were annealed and cloned into BglII and HindIII sites of pSUPER.retro.neo.GFP (Oligoengine). Real-time qRT-PCR analysis of RABL6A mRNA expression was performed with 1 µg of total RNA using a High Capacity cDNA Archive Kit (Applied Biosystems Inc., Foster City, CA) [24]. PCR reactions using specific RABL6 primers (forward 5′- aatgctgtccttcgtcatgga -3′; reverse 5′- gcacgggaaagtcatccg-3′) were performed as follows: denaturation at 95°C for 10 min, followed by 40 cycles of 95°C for 15 sec, 56.5°C for 45sec, 60°C for 1min on an ABI 7000 real-time sequence detection system (Applied Biosystems Inc.). Fold differences in RABL6A mRNA levels were calibrated to GAPDH mRNA expression and computed using ABI relative quantitation software.

### Retrovirus Production and Infection

Retroviruses encoding mouse RABL6A shRNAs, mouse ARF, human RABL6A or NPM forms were produced in human embryonic kidney (HEK 293T) cells using established protocols [24]. MEFs were transduced with sequential infections of 3 ml virus containing 8 µg/ml polybrene for 4−8 hrs (three repeats for knockdown viruses and twice for overexpression viruses). Cells were analyzed 72 hours after infection for biological effects of RABL6A loss. GFP-positivity was measured by flow cytometry (FACS DiVa instrument, Becton Dickinson) to assess efficiency of shRNA virus infection efficiency (typically >90%).

### Protein Expression and Binding Analyses

Cell pellets were lysed and protein expression levels examined by immunoblotting on PVDF membranes (Millipore, Billerica, MA) by enhanced chemiluminescence (ECL, Amersham, Buckinghamshire, UK), as described [18],[27]. In some experiments, nuclear and cytosolic fractions were obtained using a modified cellular fractionation protocol [28]. Antibodies were used to RABL6A (rabbit polyclonal made against an internal peptide [NH_2_-MVAGFQDDVDIEDQC-COOH], 1.5 µg/ml), mouse p19ARF (Novus, rat monoclonal, 1 µg/ml), NPM (Zymed, mouse monoclonal, 1:1,000), phosphorylated NPM-T199 (Cell signaling, Rabbit polyclonal, 1:1000 ), Aurora B (BD Transduction Laboratories, mouse monoclonal, 1:500), FLAG epitope (Sigma-Aldrich, M2 mouse monoclonal, 2 µg/ml), γ-tubulin (Sigma-Aldrich, clone GTU-88 mouse monoclonal, 1∶10,000), and GAPDH (Abcam, Ab8245 mouse monoclonal, 1∶10,000). RABL6A protein interactions in cell lysates were examined by immunoprecipitation-western blotting assays [18]. *In vitro* binding of *in vitro* translated RABL6A to GST-NPM recombinant protein versus GST control was performed as described [18],[27].

### Immunofluorescence and Confocal Microscopy

Three days after infection, transduced MEFs were plated on glass coverslips in six-well culture dishes (80,000 cells per well) and allowed to attach overnight. To assess centrosome numbers and RABL6A localization, cells were fixed in ice-cold methanol:acetone (1∶1) for 10 min, washed in phosphate-buffered saline (PBS), and blocked for 1 hr in PBS with 1% bovine serum albumin (BSA). Cells were stained for 1 hr with antibodies to γ-tubulin (1∶1,000), α-tubulin (Calbiochem, 1∶1,000) or RABL6A (1 µg/ml), washed in PBS and stained for 30 min with Alexafluor488 and Alexafluor568 (Molecular Probes, 1∶1,000) secondary antibodies. Nuclei were stained by 1 min incubation with 4’,6-diamidino-2-phenylindole (DAPI) in water. Samples were mounted onto slides with ProLong Antifade reagent (Invitrogen) and images captured by confocal microscopy (Zeiss LSM 710, Germany). For the HU/centrosome duplication assay, infected cells were treated for 40 hrs with 2 mM HU (Sigma) prior to fixation and staining for γ-tubulin, as described [29].

Colocalization coefficients between γ-tubulin and RABL6A at centrosomes (>50 centrosomes analyzed per sample) were determined using the Zeiss colocalization coefficient software (ZEN 2011), which utilizes the Manders overlap coefficient equation to quantify overlapping pixels. Using this software, scores can range from 0 to 1 and represent 0 to 100% co-localization within a given region, respectively. To quantify centrosome amplification, samples were examined under a 63X oil immersion lens on a fluorescent microscope (Zeiss) and the number of centrosomes (γ-tubulin positive spots) or nuclei per cell was counted for at least 200 cells per sample from 3 or more independent experiments. Statistical significance of the results was calculated using paired, two-tailed Student’s *t*-test.

### Cdk2 Immune Complex Kinase Assay

Cells (2×10^6^) were lysed on ice in NP-40 buffer (50 mM Tris, pH 8.0, 120 mM NaCl, 1 mM EDTA, 0.5% NP-40, 0.1 mM Na_3_VO_4_) for 1 hr, sonicated twice with 5 sec pulses and lysates clarified by centrifugation. Cdk2 complexes were immunoprecipitated from cell lysates using Cdk2 antibodies (Santa Cruz, sc-6248, 4 µg) and protein A/G-Sepharose for 3 hrs, and washed three times in lysis buffer and twice with kinase buffer (50 mM HEPES, pH 7.5, 10 mM MgCl_2_, 1 mM DTT). Beads were suspended in 30 µl kinase buffer containing substrate (either 2.5 µg of histone H1 [Boehringer Mannheim] or GST-NPM) plus 20 µM ATP and 10 µCi of [γ-^32^P]-ATP (ICN), and reactions carried out at 30°C for 30 min prior to analysis by SDS-PAGE and autoradiography [30]. Substrate phosphorylation was quantified by ImageJ analysis of non-saturated bands on autoradiographs from 3 or more separate experiments, and data normalized to input levels of substrate per reaction (measured by Ponceau S staining).

### Mitotic Index and Metaphase Analyses

To measure the mitotic index, infected cells were seeded (3×10^5^) into 6-well dishes and cultured overnight prior to incubation with nocodazole (100 µg/ml) for 8 hrs. Cells were collected and fixed with 70% ethanol, washed once with PBS, and incubated for 15 min in PBS containing 0.1% Triton X-100. Cells were spun down, resuspended and stained for 5 min in a DAPI solution (1 µg/ml in water) in the dark prior to dropping 10 µl of each sample onto slides for microscopic imaging and quantification (at least 200 cells quantified per sample in 3 separate experiments) [31]. Metaphase spreads were prepared from infected cells treated with Colcemid for 3 hrs, according to standard protocols [24],[32]. For quantification, blinded counts were performed on images of 40 separate metaphases for each sample. A Fisher’s exact test was employed to determine the statistical significance of differences in metaphase numbers between samples.

## Results

### RABL6A depletion results in cytokinesis failure and multinucleation

**Figure 1:**
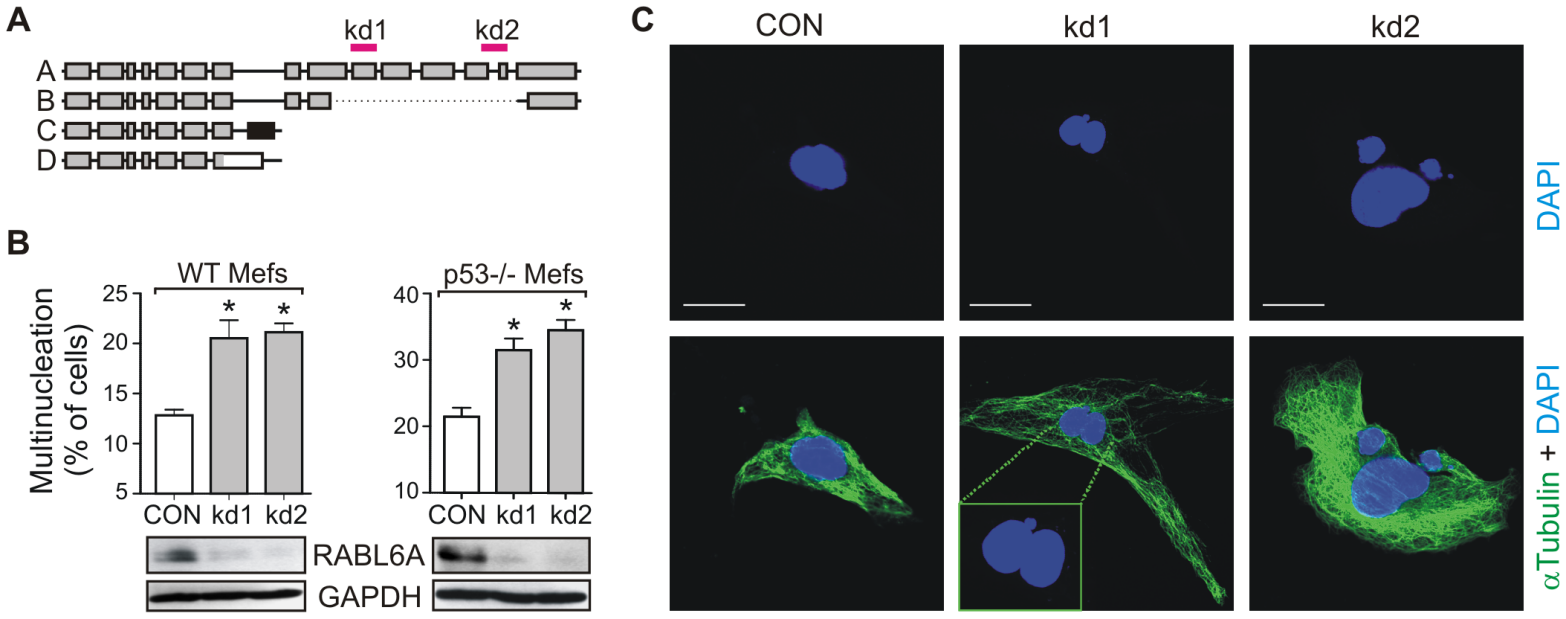
RABL6A knockdown causes multinucleation in wild-type (WT) and p53−/− MEFs. (A) Schematic of RABL6 isoforms A-D, and specificity of RABL6 shRNAs (kd1 and kd2) for RABL6A. (B) Top, Quantification of multinucleation data obtained from WT and p53−/− MEFs immunostained for α-tubulin and DAPI (nuclear stain), showing enhanced multinucleation due to RABL6A depletion. Error bars equal standard deviation from the mean from more than three separate experiments (*, p<0.001 compared to CON, as calculated using paired, two-tailed Student’s t-test). Bottom, Western blots showing effective shRNA-mediated loss of RABL6A protein in wild-type (WT) and p53−/− MEFs, with GAPDH as loading control. (C) Representative confocal images of p53−/− MEFs immunostained for α-tubulin (green) and DAPI (blue), from which data were quantified for (B). Scale bar, 20 µm.

To determine the normal biological function of RABL6, two different shRNAs (kd1 and kd2) that specifically target distinct regions of the RABL6A mRNA (Fig. 1A) were expressed in mouse embryo fibroblasts (MEFs). A scrambled shRNA (CON) was used as control, and both wild-type and p53-null MEFs were studied to assess the p53-dependence of RABL6A function. RABL6A was the focus of our analyses because it interacts with ARF and is the predominant isoform expressed in cells [18],[19]. Western blot analyses demonstrated efficient and stable knockdown of RABL6A protein in wild-type and p53-null MEFs (Fig. 1B, bottom). This was consistent with qRT-PCR analyses in p53-null MEFs showing robust downregulation of RABL6A mRNA by both shRNAs (Fig. S1). RABL6A depletion caused a significant increase in multi-nucleated, enlarged cells within both wild-type and p53-null MEFs (Figs. 1B and 1C). These data show that RABL6A is required for normal mitotic progression and cell division.

### RABL6A regulates centrosome amplification independent of p53 and ARF

**Figure 2:**
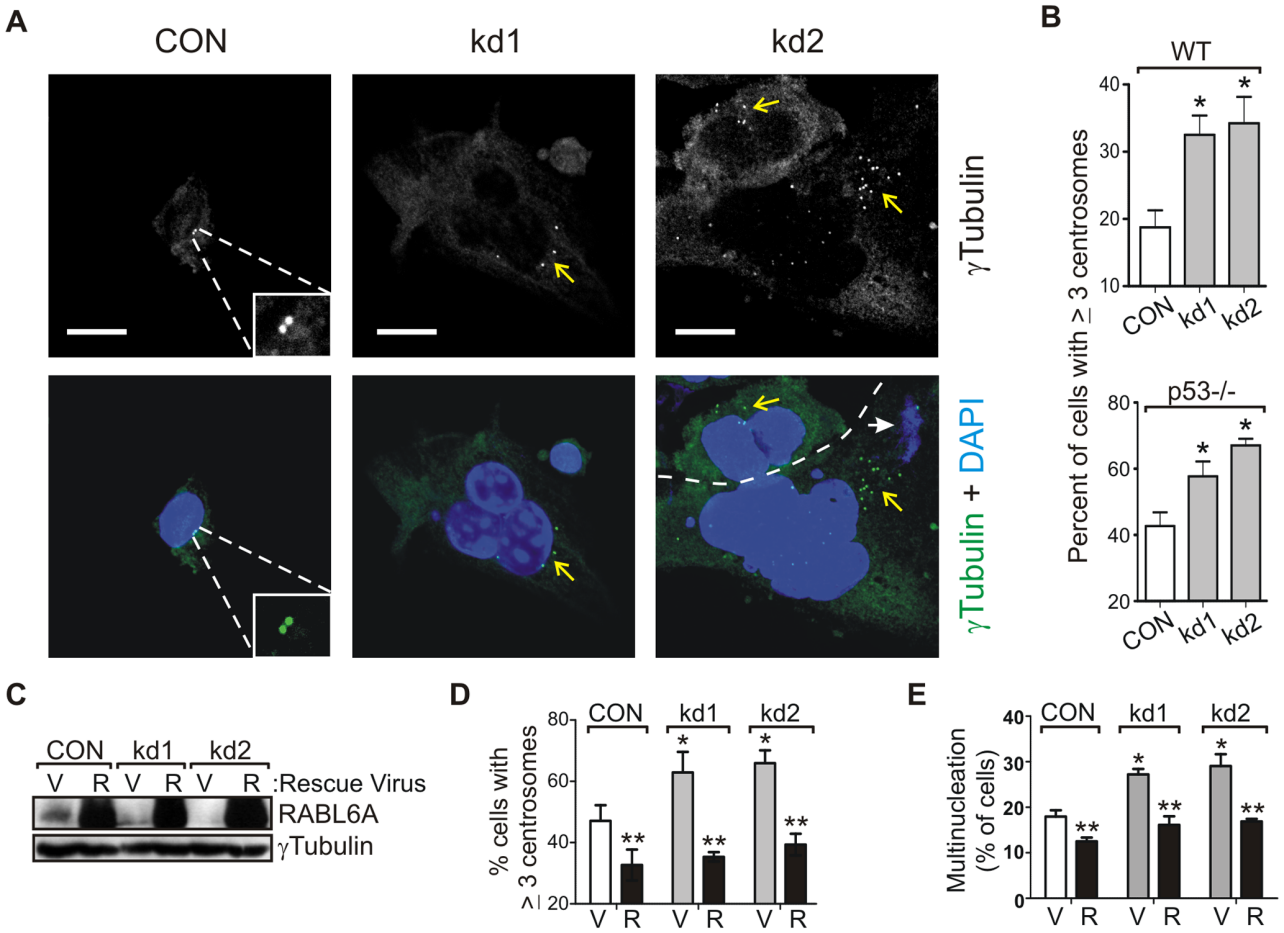
RABL6A knockdown causes centrosome amplification in WT and p53−/− MEFs. (A) Representative confocal images taken under identical settings of *p53*−*/*− CON, kd1 and kd2 cells stained for centrosomes (γ-tubulin, green) and nuclei (DAPI, blue). Yellow arrows point to areas with centrosomes, white arrows indicate micronuclei. Dashed line in the kd2 image demarcates two different cells. Scale bar, 20 µm. (B) Quantification of immunofluorescence data from (A) measuring the percent of cells with > 3 centrosomes per cell. *, p<0.05 versus CON. (C) Immunoblot showing effective mouse RABL6A depletion in p53−/− MEFs, as well as high level overexpression of human RABL6A, with γ-tubulin levels as loading control. (D and E) Quantification of immunofluorescence data for centrosome amplification (D) and multinucleation (E) in CON, kd1 and kd2 p53−/− MEFs expressing empty vector (V) or human RABL6A (R) rescue viruses. The mean values plus standard deviations were obtained from more than 3 independent experiments (*, p<0.001 compared to CON; **, p<0.01 for human RABL6A expressing cells compared to vector controls). Statistical significance for data in panels B, D and E was determined using paired, two-tailed Student’s t-test.

Multinucleation is commonly associated with centrosome amplification, both of which are present at high basal levels in cells lacking functional p53 [33]. Confocal analyses of wild-type and p53−/− MEFs immunostained with antibodies to γ-tubulin, a centrosomal marker protein, revealed that RABL6A silencing resulted in centrosome amplification in both cell types (Figs. 2A and 2B). Micronuclei formation, another sign of CIN that results from centrosome amplification, was also evident in RABL6A knockdown cells (Fig. 2A, white arrows). To verify specificity of the shRNAs, retroviruses expressing empty vector control or human RABL6A (not silenced by mouse kd1 and kd2 shRNAs) were sequentially co-infected with the scrambled control (CON) and mouse RABL6A shRNAs in p53−/− MEFs. Human RABL6A was effectively expressed (Fig. 2C) and significantly reduced the basal levels of centrosome amplification and multinucleation in CON cells (Figs. 2D and 2E). Moreover, human RABL6A completely rescued the elevated centrosome amplification and multinucleation caused by mouse RABL6A depletion in p53-null MEFs (Figs. 2D and 2E). Identical results were obtained in wild-type MEFs (data not shown). Notably, exogenous levels of human RABL6A attained in rescue studies greatly exceeded (> ten-fold) that of endogenous mouse RABL6A yet its ability to limit basal centrosome amplification and multinucleation was at most about two-fold. This suggests the effects of exogenous RABL6A on centrosome biology and mitosis are finite and saturable, as would be expected given that numerous factors control those processes. In sum, these findings reveal that RABL6A normally restricts centrosome amplification in non-transformed cells and this function is independent of p53.

**Figure 3:**
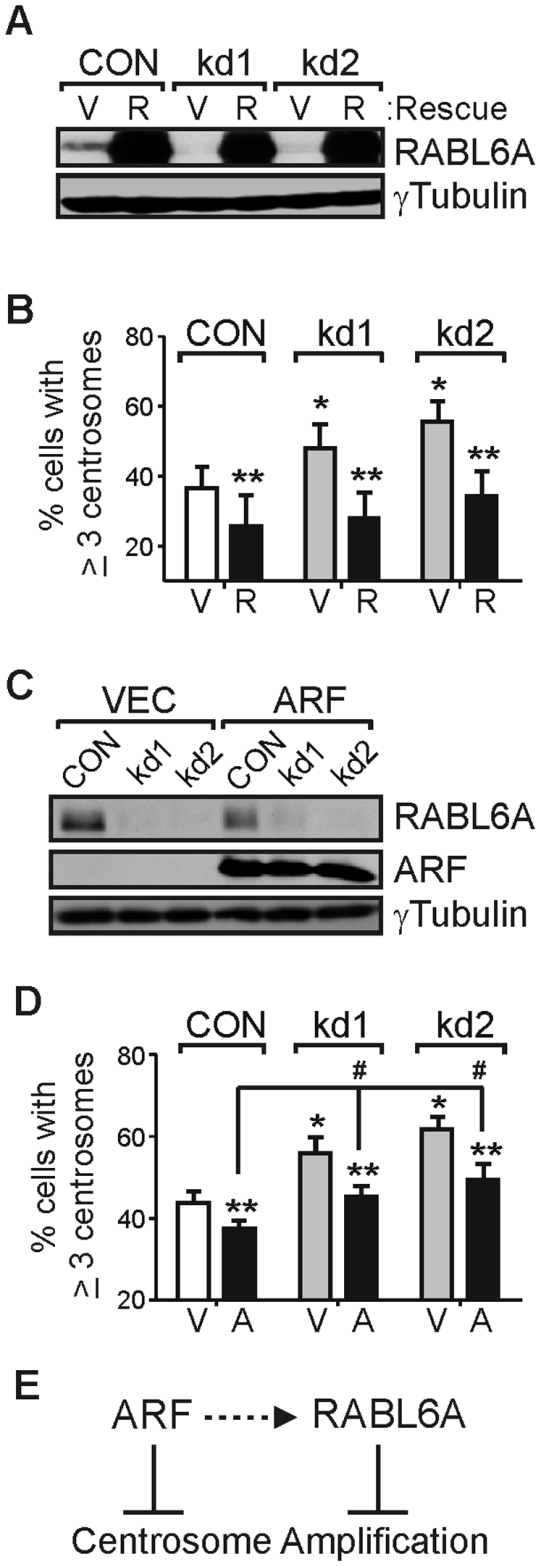
RABL6A inhibits centrosome amplification independent of ARF. (A) Immunoblot showing effective mouse RABL6A knockdown in kd1 and kd2 triple knockout (TKO) MEFs lacking *p53*, *Mdm2* and *ARF*, as well as high level expression of human RABL6A (R) versus vector (V) in CON and kd cells. (B) Quantification of immunofluorescence data for centrosome amplification in cells from (A) from three or more separate experiments. *, p<0.01 compared to CON. **, p<0.05 for human RABL6A expressing cells versus vector controls. (C) Immunoblot showing effective mouse RABL6A knockdown in kd TKO MEFs and expression of exogenous mouse ARF in CON and kd cells. (D) Quantification of immunofluorescence data for centrosome amplification in cells from (C) from three separate experiments. *, p<0.01 compared to CON. **, p<0.05 for ARF (A) expressing cells versus vector (V) controls. #, p<0.05 for kd1 and kd2 versus CON cells expressing ARF (black bars). (E) Model showing RABL6A and ARF can act separately to inhibit centrosome amplification. The dashed arrow between ARF and RABL6A indicates ARF may suppress centrosome amplification, in part, through RABL6A. Statistical significance for data in panels B and D was determined using paired, two-tailed Student’s t-test.

ARF binds to RABL6A and is a prominent tumor suppressor that can inhibit CIN independent of p53 in MEFs [17],[18]. To determine if RABL6A inhibits centrosome amplification in an ARF-dependent manner, effects of mouse RABL6A knockdown and rescue with human RABL6A were examined in p53-null cells also lacking ARF, namely triple knockout (TKO) *ARF/Mdm2/p53*−/− MEFs. Immunoblotting confirmed efficient mouse RABL6A depletion and human RABL6A overexpression (Fig. 3A). Similar to results in wild-type and *p53*−/− MEFs, RABL6A loss in TKO MEFs led to an increase in centrosome amplification that was rescued by expression of non-silenced human RABL6A (Fig. 3B). Thus, RABL6A acts independently of ARF to control centrosome numbers in cells.

It is currently not known if ARF regulates centrosome biology, although we predicted it might given its association with RABL6A as well as the centrosomal protein, NPM [27],[34],[35]. We tested if re-expression of mouse ARF in TKO cells would suppress centrosome amplification and, if so, would that require RABL6A. Figure 3C shows effective mouse RABL6A knockdown in kd1 and kd2 cells and high level ARF overexpression. ARF modestly but reproducibly reduced the basal levels of centrosome amplification in TKO CON cells (Fig. 3D). ARF also reduced the increased centrosome amplification caused by RABL6A loss (Fig. 3D). These data provide the first evidence that ARF suppresses centrosome amplification and show it can do so independent of RABL6A. Thus, RABL6A and ARF can block centrosome amplification through separate pathways (Fig. 3E). Whether or not ARF may also act, at least partially, through RABL6A to prevent centrosome amplification (Fig. 3E, dashed arrow) is currently unclear although the incomplete effect of ARF in RABL6A knockdown cells compared to CON cells suggests that is possible.

### RABL6A localizes to centrosomes

**Figure 4:**
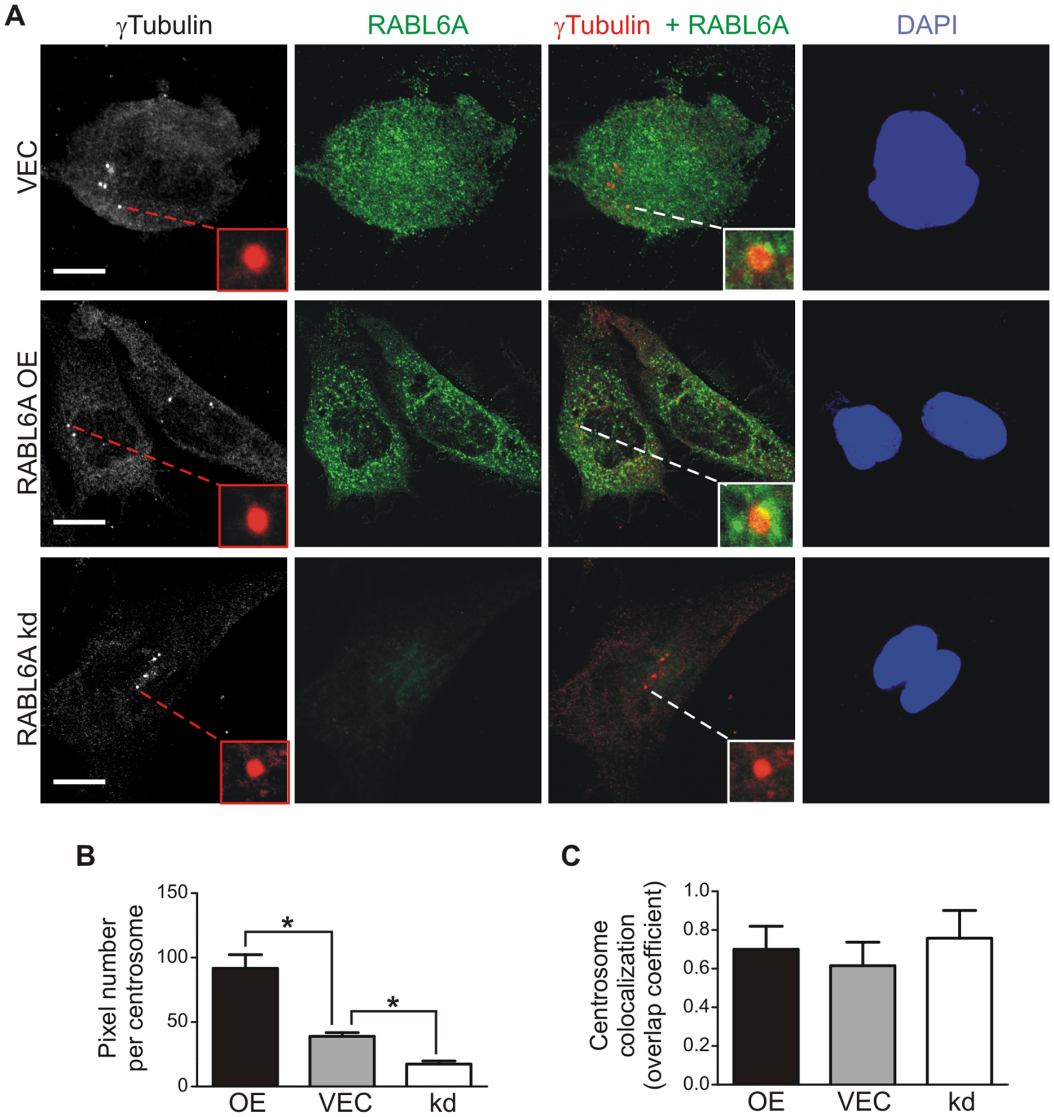
RABL6A is a novel centrosomal protein. p53−/− MEFs infected with retroviruses expressing empty vector (VEC),wild-type RABL6A (OE) or RABL6A shRNAs (kd) were stained with antibodies to RABL6A and γ-tubulin. (A) Representative confocal images show γ-tubulin (grey or red), RABL6A (green) and DAPI-stained nuclei (blue). Merged images show γ-tubulin in red with overlap between the proteins at centrosomes appearing orange to yellow. Insets magnify individual centrosomes. Scale bar, 10 µm. (B) Quantification of RABL6A-positive pixels per centrosome in cells from (A). Error bars, SEM; *, p<0.0001 as calculated using paired, two-tailed Student’s t-test. (C) Quantification of the mean weighted overlap coefficients for centrosome co-localization of γ-tubulin and RABL6A in cells from (A). Error bars, standard deviation from the mean.

Many factors controlling the centrosome localize to that organelle [1]. To determine if RABL6A localizes to centrosomes, MEFs infected with RABL6A shRNAs, empty vector or wild-type human RABL6A were stained with antibodies to γ-tubulin and RABL6A and examined by confocal microscopy (Fig. 4). We and others previously showed that RABL6A expressed in cancer-derived cell lines is predominantly cytoplasmic with variable levels found in cell nuclei [18],[19]. In vector infected p53-null MEFs, endogenous RABL6A exhibited a punctate pattern throughout the nucleus and cytoplasm (Fig. 4A). By comparison, ectopically expressed RABL6A was mainly cytosolic although like the endogenous protein it exhibited a highly punctate staining pattern. Knockdown cells expressing kd1 or kd2 shRNAs displayed greatly reduced staining for RABL6A, as expected. Notably, quantification of RABL6A-positive pixels in γ-tubulin positive centrosomes revealed that its localization at centrosomes directly correlates with its expression level (Fig. 4B). Specifically, the lowest number of pixels for RABL6A in centrosomes was found in knockdown cells, an intermediate number in vector cells expressing endogenous protein, and the highest number in RABL6A overexpressing cells. In all cases, less than 1% of the total RABL6A protein was found at centrosomes. To verify RABL6A centrosomal localization, colocalization coefficients between γ-tubulin and RABL6A at centrosomes were determined using Zeiss colocalization coefficient software (ZEN 2011), which uses the Manders overlap coefficient equation to quantify overlapping pixels. Scores of 0 to 1 represent 0 to 100% colocalization, respectively, within a given region. Analyses of over 50 centrosomes per sample revealed an average of 60−70% overlap between RABL6A and γ-tubulin at the centrosome (Fig. 4C). These findings demonstrate that RABL6A is a centrosomal protein.

### RABL6A prevents centrosome reduplication through inhibition of Cdk2-mediated NPM phosphorylation

**Figure 5:**
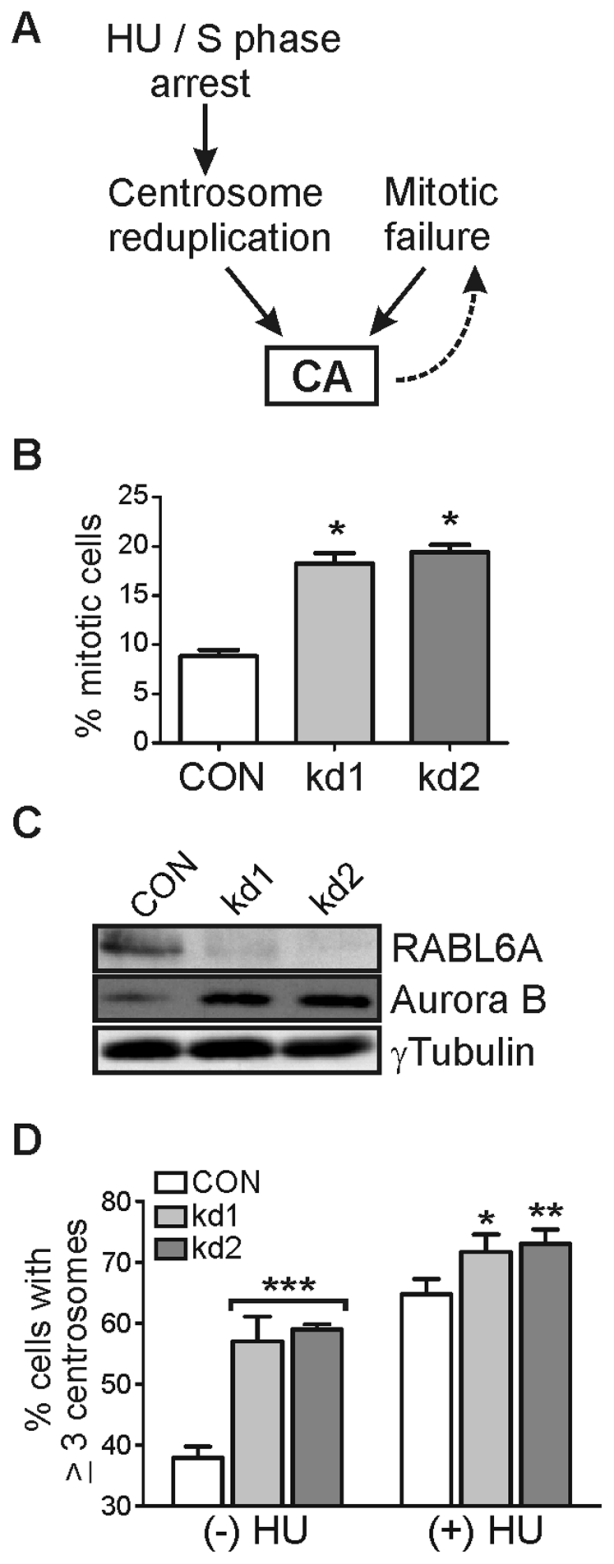
RABL6A depletion causes centrosome amplification by promoting centrosome reduplication. p53−/− MEFs were infected with CON, kd1 or kd2 shRNA viruses. (A) Schematic showing that centrosome reduplication and mitotic failure are two distinct causes of centrosome amplification (CA), whereas CA can lead to mitotic arrest and multinucleation. Hydroxyurea (HU) induces S phase arrest in cells, which in p53-negative cells leads to centrosome reduplication. (B) The percentage of mitotic cells following short-term nocodazole treatment was quantified for CON and RABL6A depleted cells. The mean and standard deviation from three separate experiments is presented (*, p<0.01 calculated by paired, two-tailed Student’s t-test). (C) Western blot analysis of RABL6A, Aurora B and γ-tubulin (loading control) showing increased levels of Aurora B in RABL6A knockdown cells. (D) Infected cells were treated without (−) or with (+) 2 mM HU for 40 hrs and centrosome numbers per cell quantified by immunofluorescence staining for γ-tubulin. Error bars represent the standard deviation from the mean from three independent experiments with statistical significance calculated using two-way ANOVA analyses (*, p<0.05; **, p<0.01; ***, p<0.001 for kd compared to CON cells).

Amplified centrosomes can be generated in multiple ways including reduplication of duplicated centrosomes or by the failure of cells to divide after mitosis (Fig. 5A). Several observations indicated RABL6A depleted cells had a cytokinesis defect, including their increased multinucleation (Figs. 1 and 2), higher mitotic index (Fig. 5B), and elevated levels of the mitotic protein, Aurora B kinase (Fig. 5C). Aurora B expression is highest in M phase [36] and its altered expression (elevated or reduced) causes failed cytokinesis, centrosome amplification and multinucleation [37]. These findings raised the possibility that failed cytokinesis may have induced the centrosome amplification associated with RABL6A loss. Alternatively, having too many centrosomes blocks cytokinesis, thus the centrosome amplification in RABL6A depleted cells could impair cell division. To resolve this “chicken or the egg” issue and determine how RABL6A depletion induces centrosome amplification, we employed a classic hydroxyurea (HU) arrest assay. HU treatment of p53−/− MEFs blocks DNA replication but allows for multiple rounds of centrosome duplication due to the absence of p53 [29],[38]. If centrosome amplification results from failed cytokinesis, HU-induced arrest in S phase will prevent an increase in centrosome amplification following RABL6A loss. This was not observed. Instead, RABL6A knockdown cells continued to develop greater centrosome amplification than CON cells in the presence of HU (Fig. 5D). These data support the conclusion that centrosome reduplication is occurring and contributing to supernumerary centrosomes in p53-null cells lacking RABL6A.

**Figure 6:**
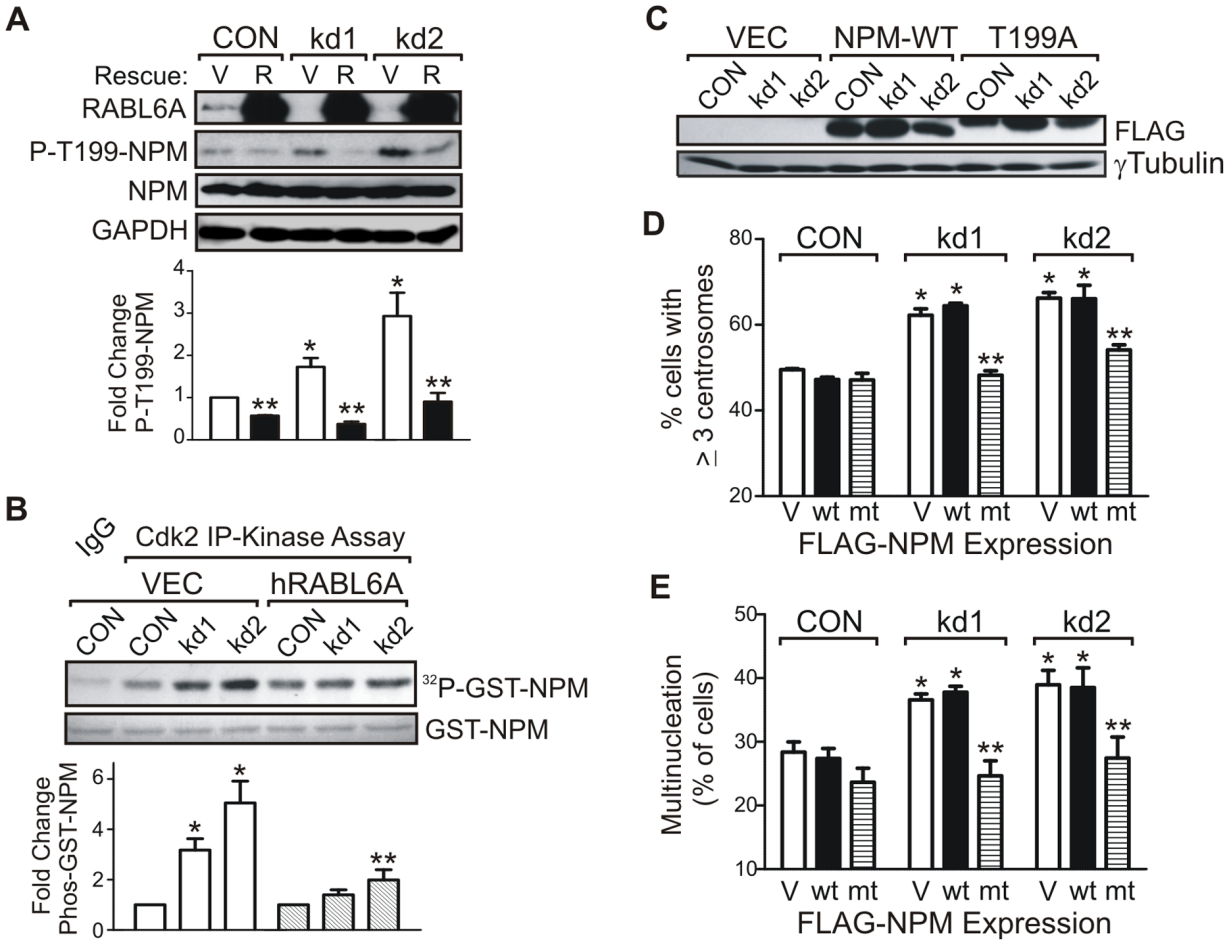
RABL6A loss leads to centrosome amplification and multinucleation via upregulation of NPM-T199 phosphorylation. (A) p53−/− MEFs expressing CON, kd1 or kd2 shRNAs were infected with vector (V) or human RABL6A (R) rescue viruses and levels of total NPM, phosphorylated NPM at T199 (P-T199-NPM), RABL6A, and GAPDH (loading control) measured by immunoblotting. The fold change in P-T199-NPM levels (normalized to total NPM) was quantified from 3 separate studies (below). *, p<0.01 compared to CON. **, p<0.05 for (R) versus (V) samples. (B) Autoradiograph of GST-NPM phosphorylation (^32^P-GST-NPM) from the indicated cell lysates following immune complex kinase assay using IgG control or Cdk2 antibodies. Graph shows the fold change in phosphorylated GST-NPM upon RABL6A loss (kd1 and kd2) or human RABL6A rescue (hRABL6A) as quantified from three experiments. * and **, p<0.01 and p<0.05 for samples relative to CON. (C, D and E) CON, kd1 and kd2 p53−/− MEFs infected with vector (VEC, V), FLAG-tagged wild-type NPM (wt) or FLAG-tagged T199A NPM mutant (mt) were assayed by western blotting (C) and immunofluorescent staining for centrosome amplification (D) and multinucleation (E). Quantified data from three separate experiments show rescue of the RABL6A loss phenotype by T199A but not WT NPM expression (*, p<0.01 compared to CON; **, p<0.01 relative to V or wt samples). Statistical significance of the data for panels A, B, D and E was determined using two-way ANOVA analyses.

Cdk2 kinase activity is required for the initiation of centrosome duplication while elevated Cdk2 activity (associated with cyclins E or A) promotes the aberrant reduplication of centrosomes [33]. NPM is a substrate of cyclin E/A-Cdk2 whose phosphorylation at T199 leads to its dissociation from centrosomes [9],[39], thereby enabling centriole separation and duplication [10]. Aberrantly high NPM-T199 phosphorylation induces unchecked centrosome reduplication during the cell cycle [33]. We found that RABL6A depletion in *p53*−*/*− MEFs caused a two- to three-fold greater phosphorylation of T199 on NPM (Fig. 6A). Moreover, expression of human RABL6A, which rescued the centrosome amplification phenotype, markedly reduced T199-NPM phosphorylation. Cdk2 immunoprecipitation (IP)-kinase assays were performed to measure the effect of altered RABL6A expression on Cdk2-associated kinase activity in cells. RABL6A depletion caused no differences in Cdk2-dependent phosphorylation of histone H1, a commonly used substrate of Cdk2 kinases (Fig. S2). However, loss of mouse RABL6A greatly increased Cdk2-dependent phosphorylation of GST-NPM *in vitro*, which was prevented by human RABL6A expression (Fig. 6B).

To define the importance of T199-NPM phosphorylation to RABL6A mediated regulation of centrosome duplication, Flag-tagged wild-type or non-phosphorylatable (T199A) forms of NPM were expressed in control and RABL6A knockdown cells (Fig. 6C). Their overexpression did not alter basal levels of centrosome amplification (Fig. 6D) or multinucleation (Fig. 6E) in CON p53-null MEFs. Wild-type NPM also had no effect on the centrosome amplification and multinucleation induced by RABL6A knockdown (Figs. 6D and 6E, black versus white bars). By comparison, the non-phosphorylatable NPM T199A mutant prevented both phenotypic changes caused by RABL6A loss (compare hatched versus white bars). These observations reveal that RABL6A specifically regulates Cdk2-mediated phosphorylation of NPM at T199. Moreover, this activity is required for RABL6A to inhibit centrosome reduplication and multinucleation in cells.

### RABL6A physically interacts with NPM

**Figure 7:**
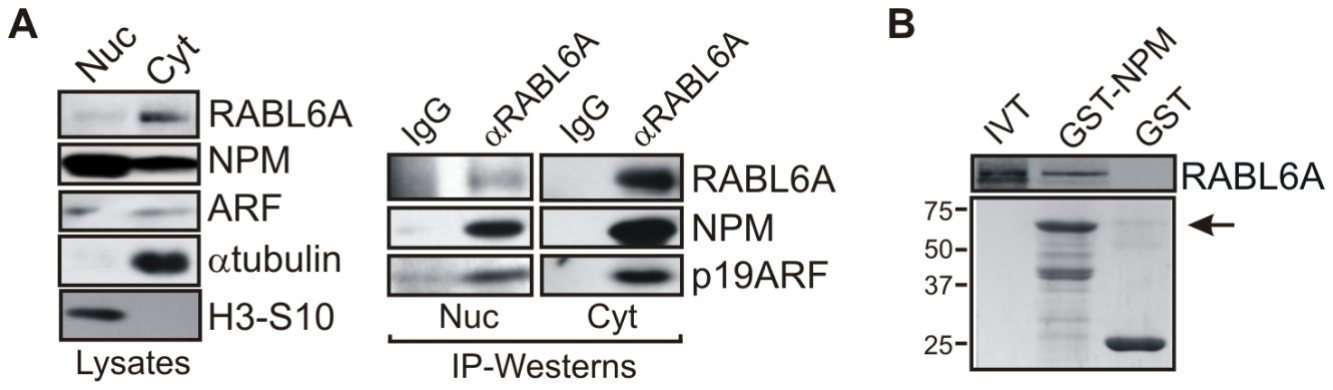
RABL6A associates with NPM *in vitro* and *in vivo*. (A) Endogenous complexes between RABL6A with NPM and ARF in p53−/− MEF nuclear (Nuc) and cytosolic (Cyt) fractions were identified by IP-Western blotting using control IgG versus RABL6 antibodies (right panel). Total protein levels in nuclear versus cytosolic fractions are shown (left), with phosphorylated histone H3-S10 and α-tubulin serving as fractionation controls for the nucleus and cytosol, respectively. (B) Top, *In vitro* binding of 35S-labeled *in vitro* translated (IVT) RABL6A to GST-NPM was detected by autoradiography. Bottom, levels of input GST and GST-NPM proteins indicated by Coomassie blue gel staining (arrow denotes full-length GST-NPM).

RABL6A binds ARF, and NPM is an important physiological partner of ARF [10],[14]. To determine if RABL6A associates with NPM in cells, IP-westerns using *p53*−*/*− MEFs were performed (Fig. 7A). Analyses of biochemically fractionated cell lysates showed RABL6A is mainly cytosolic whereas NPM, a nucleocytoplasmic shuttling protein [10], is primarily nuclear (Fig. 7A, left panel). Normally, most ARF resides in nucleoli [40] although significant fractions can be seen in the nucleoplasm [41] and the cytosol [42],[43]. p53-null MEFs express abnormally high levels of ARF with a considerable percentage detected in the cytoplasm by biochemical fractionation (Fig. 7A) and immunofluorescence (Fig. S3). IP-western data show that endogenous RABL6A (both nuclear and cytosolic) co-precipitates with endogenous NPM and ARF (Fig. 7A, right panel). *In vitro* translated RABL6A also bound to GST-NPM in the absence of other cellular factors (Fig. 7B). Together, these data demonstrate that RABL6A can directly associate with NPM independent of ARF and that endogenous complexes containing RABL6A plus NPM and/or ARF exist in cells.

### RABL6A inhibits chromosomal instability (CIN)

**Figure 8:**
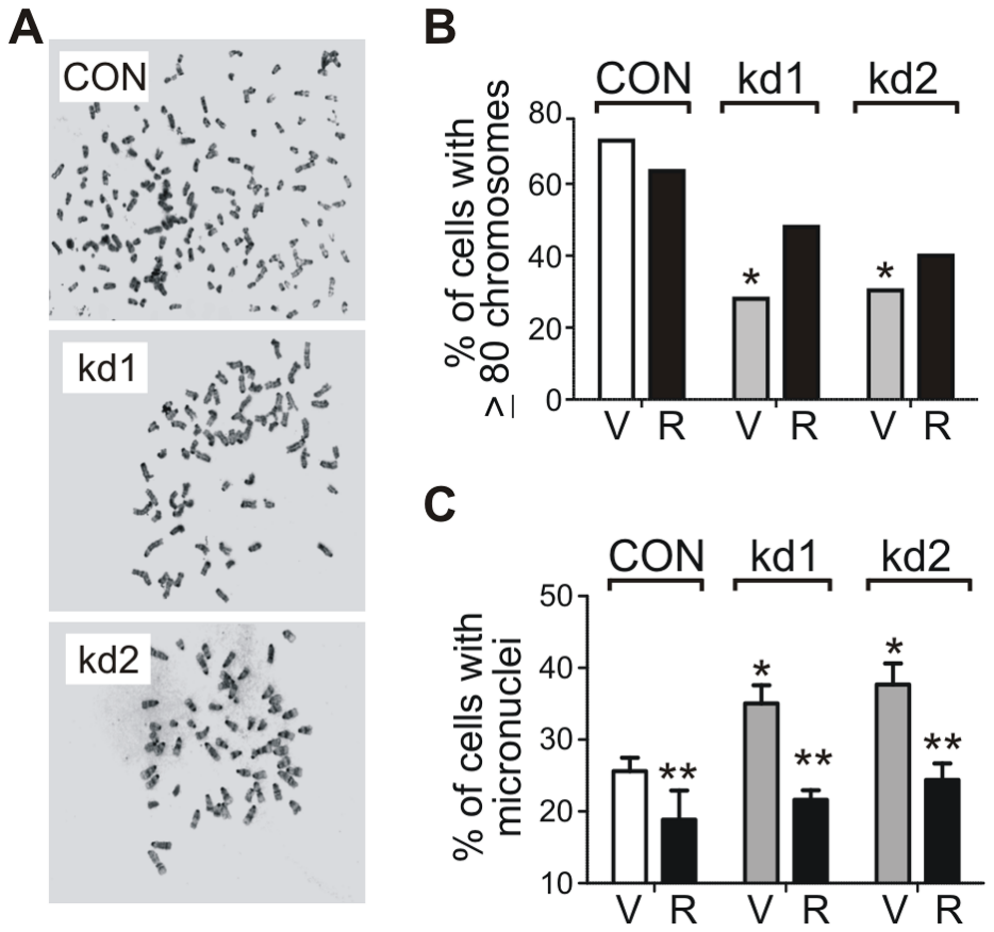
CIN is a consequence of RABL6A loss in fibroblasts. p53−/− MEFs expressing CON or RABL6A shRNAs (kd1 and kd2) were infected with vector (V) or human RABL6A (R, black bars) rescue viruses. (A) Representative metaphases of CON, kd1 and kd2 cells expressing empty vector. (B) Metaphases (n = 40) were counted and the percent of cells with >80 chromosomes graphed. Data were analyzed using a Fisher’s exact test (*, p<0.0085). (C) The frequency of micronuclei formation under each indicated condition was quantified from three or more experiments with error bars representing the standard deviation from the mean. Statistical significance was determined using two-way ANOVA analysis (*, p<0.01 compared to CON; **, p<0.05 for (R) versus (V) cells).

A defining feature of CIN is the loss or gain of chromosomes, which is a direct outcome of abnormal centrosome numbers within cells [3]. It is well established that p53-null MEFs develop tetraploidy (80 chromosomes) and general polyploidy shortly after being placed in culture [33]. Metaphase analyses revealed RABL6A loss greatly reduced the percentage of p53-null MEFs containing 80 or more chromosomes (Figs. 8A and 8B). The remarkable loss of chromosomes in RABL6A knockdown cells coincided with a significant increase in micronuclei formation (Fig. 8C), which result from chromosome misalignment and missegregation. Expression of human RABL6A partially inhibited the chromosome loss caused by mouse RABL6A depletion (Fig. 8B, black bars) and fully rescued the micronuclei formation phenotype (Fig. 8C, black bars). These results, in conjunction with the elevated centrosome amplification and multinucleation observed in RABL6A knockdown cells, demonstrate that CIN is a consequence of RABL6A loss in non-transformed fibroblasts.

## Discussion

This study examined the biological function of a new and poorly understood member of the RAB-like GTPase family, RABL6A. We found that RABL6A normally prevents centrosome amplification and maintains chromosomal stability in primary fibroblasts. In the absence of RABL6A, non-transformed cells accumulate supernumerary centrosomes and display heightened multinucleation, micronuclei formation and aneuploidy. Importantly, despite the fact that RABL6A can bind the ARF tumor suppressor [18], these activities of RABL6A were observed to be independent of ARF as well as its major downstream target, p53.

Our findings strongly suggest that reduplication of duplicated centrosomes is a primary cause of the centrosome amplification in RABL6A-depleted p53-null cells. This was demonstrated by the HU/centrosome reduplication assay and evidence that RABL6A prevents the hyperphosphorylation of NPM at T199. It is well established that controlled NPM-T199 phosphorylation by Cdk2-cyclin E/A kinases normally promotes one round of centrosome duplication per cell cycle, while Cdk2 hyper-activation causes unchecked NPM-T199 phosphorylation and extra rounds of centrosome duplication [9],[10],[33]. We observed increased NPM-T199 phosphorylation upon RABL6A loss whereas restoration of RABL6A expression reduced NPM-T199 phosphorylation and rescued both the centrosome amplification and multinucleation defects. Likewise, expression of NPM-T199A (which cannot be phosphorylated by Cdk2) also rescued the RABL6A knockdown phenotype. Thus, RABL6A prevents centrosome overduplication and consequent CIN by restricting Cdk2-mediated NPM phosphorylation at T199.

Interestingly, RABL6A was not a general inhibitor of Cdk2 activity. While RABL6A silencing enhanced Cdk2-mediated phosphorylation of NPM, it had no impact on Cdk2 activity toward histone H1. This selective regulation of a Cdk2 target rather than Cdk2 activation per se is quite novel. Other factors, such as Ras, that induce centrosome reduplication via NPM hyperphosphorylation act by increasing total cyclin-dependent kinase activity (Cdk2 or Cdk4) [8],[33],[44]. Because a fraction of RABL6A localizes to centrosomes and can directly bind to NPM, we initially thought it might reduce NPM phosphorylation by physically blocking Cdk2 accessibility to T199. However, *in vitro* Cdk2 kinase assays showed no ability of recombinant RABL6A to inhibit GST-NPM phosphorylation (data not shown), suggesting other mechanisms are involved. One possibility is that RABL6A regulates NPM localization within the cytoplasm, thereby governing NPM availability for Cdk2-mediated phosphorylation. Most RABL6A protein is in fact located in discrete foci throughout the cytoplasm, implying it plays a key role(s) in that compartment. Additional studies will be required to determine the exact mechanism(s) by which RABL6A controls Cdk2-mediated NPM-T199 phosphorylation.

How RABL6A association with centrosomes is regulated and relates to its ability to control centrosome duplication remains an important unanswered question. Like RABL6A, other centrosomal proteins, such as NPM, predominantly reside in other subcellular locations; nonetheless, NPM localization to centrosomes is essential for proper centrosome regulation [10]. Ongoing studies seek to disrupt RABL6A association with the centrosome and test if that negates its ability to regulate centrosome duplication, but so far we have not identified a RABL6A mutant that fails to localize to centrosomes. It seems likely that RABL6A localization to centrosomes is mediated by association with one or more other centrosomal proteins, possibly NPM. In addition, yeast two-hybrid studies predict that RABL6A associates with ECRG2 [45], another centrosomal protein whose loss induces the same phenotype of centrosome amplification, multinucleation and CIN that occurs upon RABL6A loss [46]. Thus, NPM and/or ECRG2 could target RABL6A to centrosomes. At present, reciprocal IP-western analyses from asynchronously growing cells have failed to support the existence of endogenous RABL6A-ECRG2 complexes (data not shown). RABL6A may associate transiently with centrosomes in a cell cycle-dependent manner, like NPM; consequently, analyses of RABL6A partners and centrosome localization at particular phases of the cell cycle may be most informative.

This study places RABL6A within a list of GTPases that control proper centrosome numbers and chromosomal stability in cells. Loss of RASSF1A or RAB6C [47],[48] or over-activation of Ras and Rho small GTPases [8],[49] induces centrosome amplification, multipolar spindles and aneuploidy. While mechanisms by which RAB6C prevents centrosome amplification are undefined, several of the other GTPases have been shown to act through pathways involving phosphorylated NPM-T199 [8],[50], similar to RABL6A. For instance, Ras promotes Cdk2 and Cdk4-mediated phosphorylation of NPM-T199 [8] whereas Rho A binds and partially activates the Rho associated kinase, ROCK II, priming it for super-activation by association with phosphorylated NPM-T199 [50]. ROCK II activation is essential and sufficient for centrosome reduplication and amplification [51]. In agreement with those reports and the elevated NPM-T199 phosphorylation caused by RABL6A knockdown, we have found increased ROCK II activity in RABL6A depleted cells (data not shown).

Previous work showed that RABL6A binds the essential growth inhibitory domains of ARF that mediate its p53-dependent and p53-independent activities [18]. Those results suggested RABL6-ARF association may be biologically important. It was therefore somewhat unexpected that RABL6A functioned independently of both ARF and p53 in our analyses. On the other hand, ARF may signal through RABL6A, at least partially. Specifically, ARF was found to inhibit centrosome amplification independently of p53 and this activity was moderately but consistently reduced in cells lacking RABL6A. As such, RABL6A may be a new downstream mediator of p53-independent ARF signaling that prevents centrosome amplification in non-transformed cells.

Centrosome amplification occurs frequently in most tumors and is thought to drive malignant progression during tumorigenesis by inducing multipolar mitoses, CIN and aneuploidy [11],[33]. The increased centrosome amplification caused by RABL6A depletion in non-transformed fibroblasts could lead to the outgrowth of genetically unstable cells and tumor formation, suggesting RABL6A may normally suppress cancer. On the other hand, elevated RABL6A mRNA levels have been observed in several malignancies, and *in vitro* knockdown studies imply that RABL6A normally promotes breast, colon and pancreatic cancer cell survival and proliferation [19]–[22]. RABL6A expression is also associated with worse outcome in breast and pancreatic cancer patients [21],[22]. Such results suggest an oncogenic or tumor promoting role of RABL6A in cancer. Indeed, we found that RABL6A is required for mitotic progression and cytokinesis, thus its overexpression could enhance tumor cell division. RABL6A may have distinct biological effects depending on cell type, cell context and/or genetic background (e.g., epithelial versus fibroblast, normal versus neoplastic, p53-positive versus p53-negative). In that sense, RABL6A may mimic the MdmX (Mdm4) protein since MdmX loss induces CIN in p53-null MEFs while its elevated expression in cancer cells and mouse models promotes tumorigenesis [52],[53]. It is also possible that dysregulated expression of RABL6A, elevated or reduced, may promote tumorigenesis. Microarray database analyses support that notion since altered levels of RABL6 mRNA, both increased and decreased, exist in a significant number of human cancers [54].

In future studies evaluating the relevance of RABL6 to cancer, it will be essential to determine the mutational status of the *RABL6* gene and relative protein expression levels of the different RABL6 isoforms in tumors versus benign tissue. While more needs to be learned about the RABL6 proteins, especially their significance and mechanisms of action in cancer and ARF signaling, the findings presented herein represent an important advance in understanding RABL6A.

## Supporting Information

Figure S1 Quantitative RT-PCR validation of endogenous mouse RABL6A silencing in p53-/- MEFs expressing kd1 and kd2 shRNAs versus scrambled shRNA control (CON). The mean and standard deviation from three separate experiments are shown (*, p<0.01 compared to CON, calculated using paired, two-tailed Student’s t-test).
(TIF)

Figure S2 Representative Cdk2 immune complex kinase assay using Histone H1 substrate. Assays were performed using whole cell lysates prepared from p53-/- MEFs expressing the indicated shRNAs (CON, kd1, kd2) with either vector or human RABL6A expression. Over multiple experiments, no significant differences in histone H1 phosphorylation were associated with mouse RABL6A silencing or human RABL6A expression.
(TIF)

Figure S3 Representative confocal images of mouse ARF (mARF) immunofluorescent staining in p53-/- MEFs (ARF-positive) versus control TKO MEFs (ARF-negative). DAPI staining of nuclei and merged images with both mARF and DAPI stains are shown. Results show that a significant amount of endogenous mARF resides in the cytoplasm in p53-/- MEFs, in addition to the expected high levels in the nucleus. Scale bar, 10 µm.
(TIF)
