# Supplementary figures and images for: RABL6A, a Novel RAB-Like Protein, Controls Centrosome Amplification and Chromosome Instability in Primary Fibroblasts
Source: PLoS One. 2013 Nov 25;8(11):e80228. doi: 10.1371/journal.pone.0080228 (PMC3839920; doi:10.1371/journal.pone.0080228)

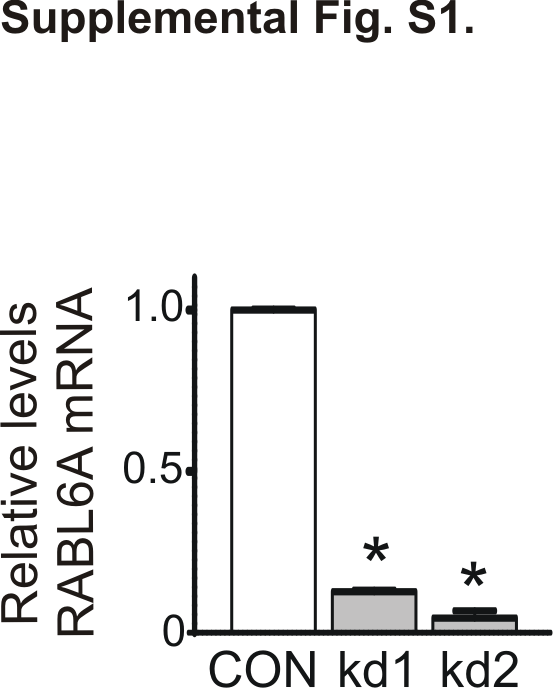

Supplement: Figure S1 — Quantitative RT-PCR validation of endogenous mouse RABL6A silencing in p53-/- MEFs expressing kd1 and kd2 shRNAs versus scrambled shRNA control (CON). The mean and standard deviation from three separate experiments are shown (*, p<0.01 compared to CON, calculated using paired, two-tailed Student’s t-test). (TIF) [file pone.0080228.s001.tif]

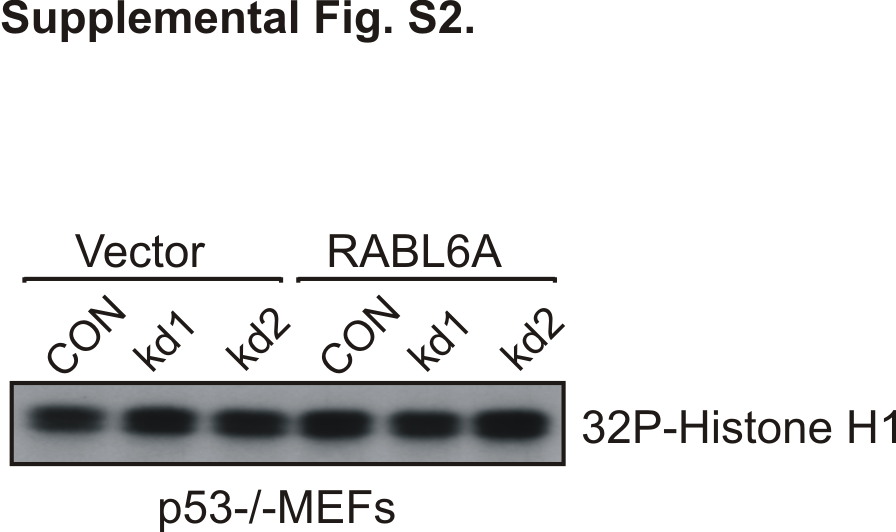

Supplement: Figure S2 — Representative Cdk2 immune complex kinase assay using Histone H1 substrate. Assays were performed using whole cell lysates prepared from p53-/- MEFs expressing the indicated shRNAs (CON, kd1, kd2) with either vector or human RABL6A expression. Over multiple experiments, no significant differences in histone H1 phosphorylation were associated with mouse RABL6A silencing or human RABL6A expression. (TIF) [file pone.0080228.s002.tif]

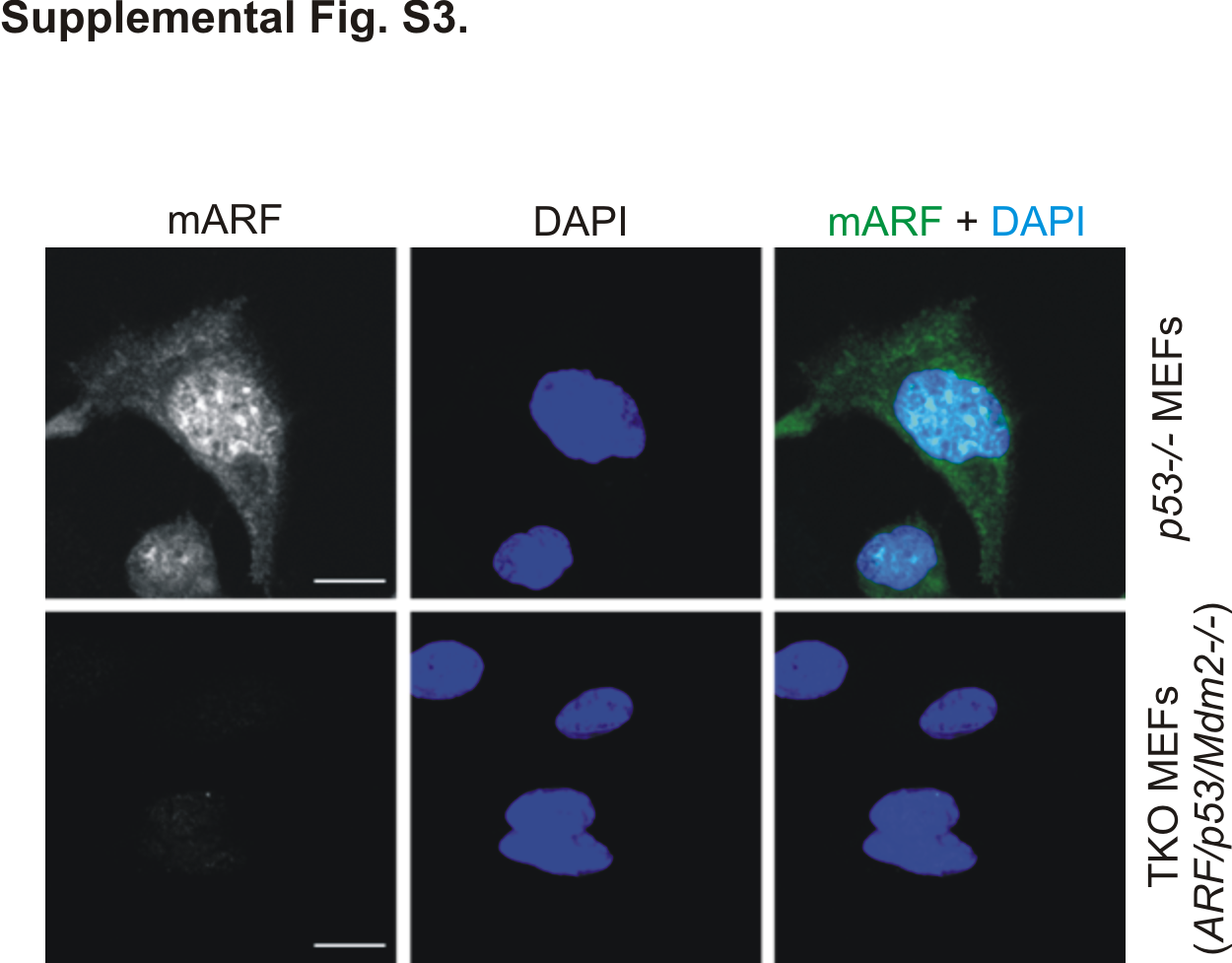

Supplement: Figure S3 — Representative confocal images of mouse ARF (mARF) immunofluorescent staining in p53-/- MEFs (ARF-positive) versus control TKO MEFs (ARF-negative). DAPI staining of nuclei and merged images with both mARF and DAPI stains are shown. Results show that a significant amount of endogenous mARF resides in the cytoplasm in p53-/- MEFs, in addition to the expected high levels in the nucleus. Scale bar, 10 µm. (TIF) [file pone.0080228.s003.tif]
